# Literature Review on Technological Applications to Monitor and Evaluate Calves’ Health and Welfare

**DOI:** 10.3390/ani13071148

**Published:** 2023-03-24

**Authors:** Flávio G. Silva, Cristina Conceição, Alfredo M. F. Pereira, Joaquim L. Cerqueira, Severiano R. Silva

**Affiliations:** 1 Veterinary and Animal Research Centre (CECAV), Associate Laboratory of Animal and Veterinary Science (AL4AnimalS), University of Trás-os-Montes e Alto Douro, Quinta de Prados, 5000-801 Vila Real, Portugal; 2 Mediterranean Institute for Agriculture, Environment and Development (MED), Universidade de Évora Pólo da Mitra, Apartado, 94, 7006-554 Évora, Portugal; 3 Veterinary and Animal Research Centre (CECAV), Associate Laboratory of Animal and Veterinary Science (AL4AnimalS), Escola Superior Agrária do Instituto Politécnico de Viana do Castelo, Rua D. Mendo Afonso, 147, 4990-706 Ponte de Lima, Portugal

**Keywords:** management, precision livestock farming, automatic milk feeding, accelerometer, infrared thermography, sound analysis, 3D camera, ruminal bolus, rumination, GPS, hearth rate monitor, partial-weight scale, machine learning

## Abstract

**Simple Summary:**

Dairy calves’ welfare is rapidly gaining long-deserved attention from science and dairy farmers’ communities. However, the elevated morbidity and mortality rates referred to in the literature reflect that there are still major problems in calves’ husbandry despite the advances already made in recent years. The development of technologies may assist the traditional time-consuming welfare evaluations and improve calves’ health and welfare on dairy farms. This review presents the state-of-the-art of technological advances related to dairy calves’ management and welfare.

**Abstract:**

Precision livestock farming (PLF) research is rapidly increasing and has improved farmers’ quality of life, animal welfare, and production efficiency. PLF research in dairy calves is still relatively recent but has grown in the last few years. Automatic milk feeding systems (AMFS) and 3D accelerometers have been the most extensively used technologies in dairy calves. However, other technologies have been emerging in dairy calves’ research, such as infrared thermography (IRT), 3D cameras, ruminal bolus, and sound analysis systems, which have not been properly validated and reviewed in the scientific literature. Thus, with this review, we aimed to analyse the state-of-the-art of technological applications in calves, focusing on dairy calves. Most of the research is focused on technology to detect and predict calves’ health problems and monitor pain indicators. Feeding and lying behaviours have sometimes been associated with health and welfare levels. However, a consensus opinion is still unclear since other factors, such as milk allowance, can affect these behaviours differently. Research that employed a multi-technology approach showed better results than research focusing on only a single technique. Integrating and automating different technologies with machine learning algorithms can offer more scientific knowledge and potentially help the farmers improve calves’ health, performance, and welfare, if commercial applications are available, which, from the authors’ knowledge, are not at the moment.

## 1. Introduction

Increasing efficiency in livestock production is needed to ensure that enough food can be produced to meet the demands of a growing population, help reduce the environmental impact, and ensure that food is available and affordable [1,2,3]. Precision livestock farming (PLF) has the potential to increase efficiency in livestock production by providing farmers with the tools they need to make data-driven decisions and optimise their management practices. This can lead to improved animal welfare, increased productivity, and reduced environmental impact [4,5]. Besides the importance of the environmental impact, technological development can improve animals’ health and welfare and farmers’ quality of life [6]. PLF employs real-time monitoring technologies to help or automatically intervene in animal management and measure individual data with time variability [7,8,9]. According to Eckelkamp [10], PLF was initially developed in poultry and swine growing operations. Applying these technologies to other farm animals is now very common, especially in more confined production systems. 

Dairy cows are considered high-value animals, and thus, applying PLF technologies at an individual level can justify the additional expenses [11]. PLF technologies applied to dairy farms have been called precision dairy farming (PDF), employing technology to measure physiological, behavioural, and production indicators to improve management and performance [12]. PLF has been more directed towards adult animals; however, increased animal welfare concerns and technology development led to an increased technology expansion for younger animals as well, such as dairy calves. In fact, according to Stygar et al. [13], future research should focus on developing and validating PLF technologies dedicated to monitoring calves and heifers’ health and welfare. Sun et al. [14] reviewed the current automated techniques to monitor calves’ health, discussing the possibility of automatically detecting less resilient calves (clinically healthy but prone to disease). 

The global trend for dairy farms is to increase in size and decrease in number, so PLF technologies can help in a manner that provides individual information about calf growth and health status. The number of papers addressing the PLF application in calves has been increasing in the last few years; however, there is a lack of agreement in several results published so far, stressing that more validation studies are still needed [9]. Nonetheless, a review and a confrontation of these results should also highlight which technologies are still in need of proper validations studies and which technologies need more field application studies in order to increase their utilisation at the farm level, where it can actively improve the calves’ welfare and hence, their performance. 

In dairy calves raising systems, technology can be implemented in health evaluations, behaviour assessments, milk, water, feed intake, and growth measurements. Nonetheless, technologies related to environmental conditions should also be considered [15], despite not being specific to dairy calves. Compared with adult dairy cows, heifers are managed in a less intensive system; however, disease prevalence is considerably high, especially in the preweaning phase [16,17]. Enterogastric, pulmonary, and umbilical disorders are dairy calves’ major health problems [18,19,20,21,22]. Although several advances have been made in providing proper treatments to ill calves, the early detection of these diseases and mortality rates are still challenging due to the absence of knowledge and, in some cases, time to monitor the animals. Nevertheless, PLF technologies that can be used as a prognosis for calves’ diseases, although lacking in validation studies [9], have been revealing promising results [23,24,25]. According to Costa et al. [9], the most researched PLF technologies for managing the performance and health of pre-weaned dairy calves are triaxial accelerometers, automatic milk feeding systems (AMFS), and technology that measures physiological or physical attributes, such as infrared imaging and 3-dimensional cameras.

In this review, we explore and debate recent advances in the most conventional PDF technologies, such as triaxial accelerometers and AMFS, but also in other technologies whose application in calves has not yet been extensively reviewed, as well as the simultaneous integration of different technologies and machine learning techniques.

## 2. Technological Applications to Monitor Calves’ Health and Welfare

Current technology research to monitor and manage dairy calves is mainly related to health disorders and painful procedures. However, the literature addresses that good animal welfare should not be viewed as merely the absence of pain, discomfort, or hunger but by the quality of life and emotional states. According to Fraser [26], animal welfare should contemplate three views: essential health and functioning, natural adaptations, and affective states. Near future research must also focus on evaluating other welfare standards, such as the absence of fear and the presence of positive emotional states. A good example is a PhD thesis published in 2021, where the positive and negative emotions of calves were studied with infrared thermography by analysing temperature asymmetries in different regions of interest, such as the difference in temperature between the left and right-side hemispheres [27]. Studying farm animals’ emotions would allow us to better understand how these animals perceive and interact with the environment [27], including their interaction with humans. In this way, a better quality of life could be achieved if the economics and social aspects inherent to animal production could be overcome. 

Although more studies are still needed in this area, noninvasive precision techniques to access animals’ emotional states can provide the knowledge needed to improve farm animals’ welfare. Studies with calves, AMFS, and triaxial accelerometers make up the majority of the literature in this area and have already been properly reviewed [9]. However, there are other emerging technologies and different approaches that have not yet been extensively reviewed, e.g., 3D cameras for body measurements and IRT to evaluate acute and prolonged pain situations. One of these recent approaches, which has been gaining popularity, is the simultaneous integration of different technologies to monitor and evaluate the calves’ health status, performance, and pain since it can provide data from different sources, which, in turn, measures different physical, physiological, and behavioural traits. Usually, this multi-technological approach generates much more data that can be more easily processed using machine learning and deep learning techniques rather than traditional statistics. 

### 2.1. Automatic Milk Feeding System

Automatic milk feeding system (AMFS) is probably the most extensively used technology in dairy calves, which can significantly increase the efficiency of dairy calves’ raising systems [28]. AMFS can feed multiple calves several times per day without increasing labour requirements [28]. Calves should be fed several times a day since excessive quantities of milk provided at once can increase the risk of dietary diarrhoea. In the AMFS, the daily dose is distributed throughout the day, so higher milk feeding levels can also be achieved compared to the manual system. It has been demonstrated that increasing milk allowance with this strategy can improve calves’ performance [29,30,31]. AMFS also makes it possible to do automated gradual weaning, which contributes to minimising post-weaning stress and thus achieving better performances [32]. 

**Figure 1:**
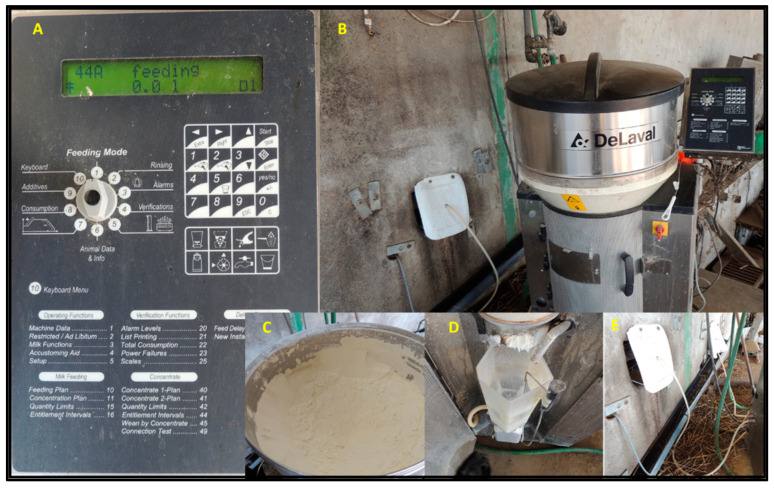
Automatic milk feeding system: (**A**) shows the control panel, where all the information relative to the calves and the machine is registered and controllable; (**B**) shows all the components (except the RFID reader and the teat, which are inside the calves’ barn); in (**C**) shows the milk powder deposit; (**D**) is the mixer cup, where each portion of milk is prepared with milk powder and heated water; in (**E**) is shown the circuit in which the milk departures from the mixing cup to the teat inside the calves’ barn.

To function with the AMFS (Figure 1), an electronic identifier must be attributed to each calf. Then, relevant information from the calf can be registered in the AMFS software, such as the birth date and body weight. Then, a program regarding milk allowance per day and days being fed is selected for each animal. During the selected period, the calf can drink a certain amount of milk distributed throughout the day. To provide a more distributed feeding throughout the day, the AMFS blocks consumption between consecutive visits, predefined in the software. The AMFS software records individual information about the drinking behaviour of every calf with an active responder. However, the maximum number of calves per unit depends on the machine’s capacity. So, it is crucial to be aware of the machine’s functionality, such as calibrating milk powder doses, checking for the thermostat accuracy, water flow, etc. 

Like other automatic monitoring technologies, feeding calves through a robotised system can decrease human-animal interactions, which may not be favourable [33]. An increase in the physical distance between the farmer and the animal could lead to animals becoming more reactive when handled [33]. Older calves (without an active responder) may usurp the younger calves’ portion of milk by means of displacement behaviours when groups are not properly managed. The cost of acquisition and maintenance of the machine, which may not be accessible to every farmer, can also be a downside. Nevertheless, AMFSs are well recognised for providing helpful information to monitor calves’ health, performance, and welfare [9]. 

Daily milk intake, rewarded and unrewarded visits to the feeder, and drinking speed are the three most studied indicators. The use of AMFS to predict the onset of diseases and to evaluate welfare has been successfully tested [24,25,28,34,35,36,37]. Sick calves showed less milk intake, lower drinking speed, and a decrease in unrewarded visits from −2 to −4 days before diagnosis [23,24,34,36,37] that varied with milk allowance level [25,35]. Calves fed with higher milk volumes tend to change their feeding behaviour [25,35]. Cantor et al. [38] used AMFS alarms triggered when the calf reduced 20% of the average milk intake and 30% of average drinking speed based on the past 12 d mean to evaluate the administration of colostrum replacers in health and performance improvement. Conboy et al. [39], in a cross-sectional study involving 523 observations, reported a 63% reduction in daily milk intake of calves with bovine respiratory disease (BRD) and 57% in calves with neonatal calf diarrhoea (NCD). In another study, calves with NCD had a significative decrease in milk intake on the day of diagnosis, 5 days after (observed daily by faecal consistency), and a tendency for −1 d (*p* = 0.09); as for rewarded visits, diarrheic calves presented fewer visits at −1 d and 0 d than healthy calves, but more visits at 3 d and a tendency at 4 d [40]. Moreover, the parallel sensitivity and specificity on the day of diagnosis using milk intake and rewarded visits were not satisfactory (Se of 69% and Sp of 22%). The authors of this study reported that data from the AMFS could not replace the identification of calves with NCD through clinical examination. 

One of the animal welfare’s “Five Freedoms” is “Freedom from hunger and thirst”; AMFS indicators may help provide information about prolonged hunger in preweaning calves [29,30]. AMFS may also be used to study calves’ preferences for milk composition. For example, it was shown that calves had a preference for whole milk over milk replacers [41]. AMFS information was also used to associate temporal between individual differences in feeding behaviours with personality traits, highlighting two distinct groups: calves that visited the machine more often had a superior drinking speed and grew faster, and calves that visited the machine fewer times had an inferior drinking speed and grew slower (sick calves were excluded from the analysis) [42]. Nevertheless, it should be noted that calves can present a distinct individual behavioural rhythmicity throughout the day [43].

### 2.2. Triaxial Dimension Accelerometers

An accelerometer measures proper acceleration, given by the body’s velocity per unit of time in its instantaneous rest frame. Triaxial accelerometers (3D accelerometers) measure proper acceleration simultaneously in three orthogonal directions (x, y, and z) at regular time intervals (10 to 20 Hertz seems to be the most efficient frequency [44]). The generated data can be stored in a data logger or a smartphone or used raw with a commercial system applied [44]. Three-dimensional accelerometers provide information in a three-dimensional plane, unlike unidirectional accelerometers. It records the acceleration forces produced by the animal movement, which are associated with certain behaviours (e.g., lying, standing, suckling, ruminating, and chewing), allowing automated real-time monitoring of the animal’s activity, health, and welfare when combined with a proper algorithm. However, certain behaviours are more difficult to predict than others (e.g., standing up and lying down), decreasing the prediction accuracy of these behaviours [44]. In dairy farms, accelerometers are well established as a valuable tool to provide information about the oestrus cycle in cows [45,46,47]. However, practical application in dairy calves is still limited, being used only for research purposes. The battery drainage can also be a limitation, which is related to the sampling frequency and can go from 5 days [48], 15 days [49], to 2 years [50].

The use of 3D accelerometers has been used for a variety of topics within calves’ welfare evaluation. Accelerometers have a fundamental basis for behaviour analysis, such as lying and locomotor behaviours. Gait pattern analysis was first successfully achieved with 3D accelerometers by de Passillé et al. [51]. Nevertheless, lying behaviour is regarded as a primary indicator for welfare measurements [24,25,37,52,53,54,55,56,57,58]. The lying time duration and the number and duration of lying bouts can be important indicators of calves’ health and welfare status [59]. Manual record of these events has low time-labour efficiency and thus cannot be performed daily. Three-dimensional accelerometers provide a noninvasive measure of calf behaviour, using algorithms to process natural position, speed, and directional data [9]. Data acquired with accelerometers have the potential to provide information for disease detection before illness onset, measure pain behaviours more objectively, and evaluate positive welfare situations. 

According to Ahloy-Dallaire et al. [60], play behaviour, such as jumping, running, and kicking, are good indicators of improved animal welfare. According to Luu et al. [61], an accelerometer provides reasonable estimates of play behaviour in calves, reducing the sampling rate and measuring acceleration only in the vertical axis. Conversely, Größbacher et al. [62] recorded accelerometer measurements at 1 Hz (in 1-s intervals) and showed that the vertical axis could not be used alone to quantify absolute levels of calves’ locomotor play in the home pen. Interestingly, Größbacher et al. [63] found, using accelerometers, that negative play among calves has a contagious effect. Friesian male calves (*n* = 325) were monitored with an activity-monitoring device (Fedometer system, FEDO; ENGS, Rosh Pina, Israel) from 30 to 90 days of life, recording the number of steps, number of lying bouts, and lying time. Frequency and time of visits to the feed bunk were monitored with a proximity sensor placed in the feed bunk; prior to ten days of illness, sick calves’ behaviour changed, with fewer steps, less number of visits and lesser time in the feed bunk and increased lying time [58]. However, the best prediction model was only at −1 day prior to disease with a moderate accuracy of 71.5%, Se of 68.8% and an Sp of 72.4%. Gardaloud et al. [64] reported a predictive model for BRD using behaviour data (obtained with an ear-attached 3D accelerometer) from −2 d and −3 d before symptoms with better Se (71.4%) and Sp (95.2%). Moreover, in Goharshahi et al. [65], diarrheic calves had a longer lying time (64.8 min) −1 day prior to clinical identification compared with control calves (ear tag-based accelerometers; Smartbow GmbH). Similarly, calves with BRD spent more time lying than healthy calves on day −1 [66]; however, the lying bout duration was already greater on day −2 (leg-mounted Axivity AX3). In contrast, in Lowe et al. [24], there were no significant differences in lying time before clinical signs of disease in calves experimentally infected with rotavirus. However, the number of lying bouts decreased, and the duration of the lying bout increased (leg-mounted Hobo Pendant G data loggers).

With an accelerometer placed in the left ear (Smartbow, GmbH, Weibern, Austria), positive results were obtained in detecting lying behaviour, rumination, feed intake, and other activities in calves [56]; however, further development of the algorithm was mentioned as necessary to produce reliable results for milk and water intake. The CowManager Sensor was tested in calves to measure feeding and rumination behaviours against visual observation [67]. The feeding behaviour was well correlated with visual observation (r = 0.88), but rumination was not (r = 0.63). 

Monitoring of negative welfare can also be accessed with accelerometers, for example, in painful procedures such as disbudding [68,69] and castration [70]. The ideal scenario was that these technologies could automatically collect all the behaviours traditionally collected by visual assessment, either in person or with video recordings, and other behaviours that are difficult to measure, such as the number of steps manually. However, the problem seems to lie in a lack of agreement on which indicators are more suitable for pain behaviour analysis. For example, the calves’ cautery was disbudded with or without anti-inflammatory administration (flunixin meglumine) and did not show differences in behavioural parameters measured with 3D accelerometers (Hobo Pendant G data logger, Onset Computer Corp.) attached to the right hind leg [71]. Cornual nerve block was performed in both groups, which could explain the absence of statistical differences, although differences in cortisol levels were reported [71]. In a similar study, calves under the effect of anti-inflammatory (meloxicam) were less active than control calves during the first 5 h following dehorning [72]. Nonetheless, lying behaviour (measured with the same 3D accelerometers from the previous study) did not differ between calves’ sham disbudded and disbudded using caustic paste with (one group with meloxicam only; another group with lidocaine, and the other with meloxicam + lidocaine) or without pain control; although other indicators of pain presented statistical differences between groups [69]. Dehorned calves without local anaesthesia but with anti-inflammatory injection (meloxicam) or without anti-inflammatory (control) had equal lying times. However, control calves had less lying time post-dehorning than predehorning [73].

Depending on the painful procedure, animals can express a different behavioural response. In a study with beef calves, all of them reduced the number of steps during a 24 h period after castration in comparison with the period prior to castration, regardless of pain control; however, only the calves without pain control presented statistical differences [74]. A dose of 1 mg/kg BW of meloxicam oral suspension was able to reduce the display of painful behaviours and physiological responses after castration (band and surgical methods); calves treated with meloxicam had greater activity and less lying time and number of lying bouts than nontreated calves [75]. The stride length measurement has been used to assess post-castration pain in calves [74], but it seems to not be substantiated [70,76].

### 2.3. Infrared Thermography

Abnormal body temperature is usually related to illness, and IRT can detect this alteration [77] by measuring radiated electromagnetic energy [78]. IRT measures the animal’s surface temperature and thus provides information about some physiological aspects. Usually, thermographic images are taken in highly vascularised anatomical regions, known as thermal windows (e.g., orbital, nasal and perineal regions [79]). However, other regions are also used in IRT validation studies, such as the scapular, the masseteric, the sacral, the umbilical, and the scrotal regions. The orbital region temperature measurement has already been shown to have good intra- and inter-repeatability with IRT in calves under 12 weeks of age [80]. However, there are no similar studies with calves for other anatomical regions. The number of studies with calves using IRT has been increasing in recent years [20,24,25,70,81,82,83,84]. According to Costa et al. [9], the actual temperature of body locations such as eye, shoulder, ear, or side is unknown in calves since IRT measures radiant heat, so IRT is almost exclusively used in illness or pain assessment. 

IRT can be used as a noninvasive diagnostic tool providing information to predict infections in calves before clinical symptoms are detected [85]. However, so far, IRT applied to calves has only been used to diagnose enteric diseases, respiratory diseases, and umbilical inflammation. 

From the author’s knowledge, IRT was first validated in calves as an early indicator of bovine viral diarrhoea [85]. Ten of fifteen heifers were inoculated with bovine viral diarrhoea virus at approximately six months of age, and different body regions were monitored with IRT. The authors have found that the infrared temperature of the orbital region was the most sensitive parameter, with a significant increase one day after inoculation (lower than 1 °C). These changes occurred up to one week before confirmation by laboratory tests, such as haptoglobin levels [85]. Later, Lowe et al. [24] also found an infrared temperature alteration in dairy calves with NCD, infected with Salmonella. In this study, the temperature of the lateral region increased; the shoulder (over the trapezius) decreased before clinical signs of disease were detected (up to 4 d before), showing a higher sensitivity than the orbital region, which decreased, but only when the average of the 7 days after and before clinical signs was compared. According to the authors, the temperature increase in the lateral region was probably due to the proximity to the site of infection and localised inflammation of the intestines, and the decrease in temperature of the shoulder could have been due to a restriction of blood flow to the extremities, a response originated from a fever state [24]. Contrary to Schaefer et al. [85], a significant change in the orbital temperature was not observed prior to clinical signs [24]. The metabolic status of the calf influences the heat balance as well as the amount of dissipated heat at the animal’s surface so that it can interfere with IRT readings. A significant drop in cheek and ear infrared temperature was observed prior to a diarrhoea bout in calves fed 5 L/d milk but not on calves fed 10 L/d milk [25], showing that milk allowance can affect the IRT capacity as an early predictor of disease. The authors suggested that the drop in temperature may be due to a diminished metabolic rate in the 5 L/d milk group caused by less energy intake, which did not occur in the 10 L/d milk group [25]. The relation between calves’ infrared temperature of different body regions and enteric diseases is still not fully understood and needs to be further evaluated. Moreover, it can be suggested that pathogenesis and different etiologic agents can have a significant role in the thermogenesis of the calf and, consequently, in the IRT results. 

IRT was studied in calves as an early indicator of BRD [82,86,87]. An increase in the orbital temperature was observed 4–6 days prior to the onset of clinical symptoms in weaned calves [86]. The best sensitivity (68.7%) and specificity (77.4%) were achieved with IRT mean ratio (the mean infrared temperature for the animal divided by the mean maximum temperature for the contemporary group). An automatic system combined with radio-frequency identification (RFID) placed near a water point could identify calves positive or negative to BRD through the infrared temperature of the orbital region [82]. Calves positive to BRD showed a higher peak infrared temperature (35.7 ± 0.35 °C) compared to calves negative to BRD (34.9 ± 0.22 °C) [82]. From the authors’ knowledge, there are no further works using IRT as a tool to monitor BRD in calves and to provide validation to these studies. In a narrative review published in 2021 addressing predictive models for BRD in feedlots, IRT was not well placed, mainly due to extra training and costs compared with other methods [88] and a lack of validation studies. Abnormal respiration is a symptom of BRD, so the automatic evaluation of the respiratory frequency could be a valuable tool. Lowe et al. [89] used IRT (T650sc; FLIR Systems AB, Danderyd, Sweden) to monitor the respiratory rate in five dairy calves. A high coefficient of determination (R^2^ = 0.93) between counting flank movements recorded with a video camera and thermal fluctuations around the nostrils during inhalation and exhalation was obtained [89]. Besides the BRD application, this technique could also be interesting in evaluating tachypnoea associated with heat stress. 

Omphalitis is another common prevalent disease in dairy calves, which is diagnosed based on inflammation signs in the umbilical region [90]. However, some calves can present intra-abdominal inflammation without external signs of inflammation, which makes early detection crucial for the calves’ welfare. Few studies address IRT as a tool to diagnose omphalitis in calves, and they have not been reviewed to the author’s knowledge. A thermographic camera (FLIR SYSTEMS AB, Sweden; Model: T620 25°) was used to evaluate the efficacy of IRT on the diagnosis of omphalitis in calves up to 30 days of age [20]. Calves diagnosed with omphalitis by physical examination had a significantly higher maximum temperature in the lateral umbilical region than calves clinically healthy (37.0 ± 1.1 °C for the omphalitis group and 35.7 ± 1.8 °C for the control group; *p* = 0.002) [20]. In Robinson et al. [91], a portable infrared thermometer (Dual Laser 50 model 42570, Ex-tech Instruments Corporation, Waltham, MA, USA) was used to measure the surface temperature of the umbilical stump as an indicator of infection, but none of the calves presented signs of infection. Steerforth and Van Winden [90] proposed a scoring system for the diagnosis of omphalitis, which includes temperature measurement of the umbilical region with IRT. In the multilinear regression model for omphalitis, umbilical cord infrared temperature was the smallest contributor to the model (β = 1.3, *p* = 0.003), which included discharge (β = 4.14, *p* < 0.001), umbilical hernia (β = 1.73, *p* < 0.024), and swelling (β = 1.72, *p* < 0.001. However, only discharge and infrared temperature of the umbilical region were significantly associated with intra-abdominal inflammation in the univariable logistic regression analysis (*p* = 0.031). The authors found that an increase in 0.5 °C of the umbilical stump temperature above a sternal reference temperature had an OR of 2.90 (95% CI: 1.10; 7.63) for intra-abdominal inflammation [90]. These results suggest that IRT may have the potential to detect intra-abdominal omphalitis before the occurrence of topic signs, such as the presence of purulent discharge. Nevertheless, when addressing omphalitis as a general inflammation of the umbilical region, other signs such as discharge, swelling, and umbilical hernia may be enough for the trained professional. Another hypothesis yet to be tested is if the infrared temperature of the umbilical region increases prior to clinical diagnosis. Furthermore, since pain behaviours are associated with omphalitis, it could be interesting to test if 3D accelerometers can identify and predict calves prone to this disease. 

Pyrexia is a common symptom associated with infectious diseases, and its evaluation is integrated into most diagnostic protocols. Pyrexia in calves is commonly assessed through rectal temperature; however, the studies correlating rectal temperature with IRT have poor to moderate results. Bell et al. [92] found a weak correlation between the thermal image temperature of the eye and rectal temperature with a thermal camera in preweaned calves (r = 0.28; FLIR SC620, FLIR Comp, Boston, MA, USA). Cossa et al. [93] found a moderate correlation (r = 0.50) between the infrared temperature of the eye and the rectal temperature of Holstein’s calves. Scoley et al. [80] obtained weak correlations between the calves’ core body temperature (rectal temperature) and the infrared temperature of the eye and the rectal area (correlations ranging from 0.16 to 0.47). Cantor et al. [94] also tested the infrared temperature of the orbital region against the rectal temperature in 318 male Holstein calves, but the results substantiated the previous findings. From these results, IRT is not a viable technique to diagnose pyrexia, which can be easily performed by measuring rectal temperature with a thermometer, despite the practical advantages of an automated system that could identify fever in group-housed calves. An accurate but more invasive measure of core body temperature could be accomplished with temperature microchip implants, although its accuracy differed with the implant site [95]. IRT showed promising results in evaluating vaccination’s safety and local effects in calves [96]. However, more studies are needed to corroborate these results, as it is the first study using IRT to evaluate the vaccination’s local effect on calves.

From the current studies, IRT can be a viable technique to diagnose respiratory and enteric diseases and local inflammatory processes in calves since it is sensible to radiant heat emitted in locally affected areas but not to variations in core body temperature.

Pain control during pain management procedures such as disbudding, dehorning, and castration is extremely important to secure the calf’s welfare. The efficacy of anaesthetic and analgesic drugs administrated during these procedures has been gaining interest in recent years [97]. IRT can be used as a tool to evaluate these pain procedures since alterations in the sympathetic nervous system can be perceived by orbital region temperature fluctuation due to a redirection of blood flow to skeletal muscles [98,99]. Most of the studies that use IRT to measure pain in dairy calves have focused on disbudding [100,101,102,103]. 

Disbudding is a painful procedure that is executed in most dairy farms, leaving wounds that can take 9 weeks to heal [104]. It is hypothesised that, due to autonomic nervous system response, a decrease in the temperature of the eye occurs upon pain and/or stressful events [105]. Stewart et al. [81] was the first published paper evaluating the capacity of IRT as an index of pain in calves. This work showed that the temperature of the eye abruptly decreased immediately after disbudding (with a gas-powered cautery iron) without local anaesthetic, in contrast to the nonsignificant decrease in calves disbudded with a local anaesthetic (6 mL of 2% lignocaine hydrochloride). A possible reason is that sympathetic vasoconstriction occurs in response to a stressful stimulus [81]. Ten minutes after the procedure, the temperature increased above the baseline in the disbudded groups (with or without anaesthesia). In the control group (sham disbudding), there was no significative alteration in the eye temperature. 

Castrated calves also experienced an increase in the maximum eye temperature 5 to 20 min after the procedure (both calves castrated with or without anaesthesia —lignocaine hydrochloride [106]). Kleinhenz et al. [107] observed that castrated calves 2 h after castration with flunixin administration had increased maximum eye temperatures, compared with calves castrated without anaesthetic and sham castrated; however, it was not indicated the level of significance between treatments. The change in temperature of the eye was also tested as a pain biomarker in calves upon disbudding (electric-powered cautery) with or without the administration of an analgesic (flunixin meglumine). However, no differences were found between both groups in any of the pain biomarkers studied, except for cortisol [100]. A decrease in the maximum temperature of the eye was observed in all calves at hours 1 and 2 after the procedure. However, according to the authors, it was not an indication of a stress response, and the time of the first measurement and environmental temperature could have affected the results [100]. In fact, in Stewart et al. [81], the drop in temperature was only within the first minutes after disbudding, with a gradual increase until 15 min above the baseline. Adcock and Tucker [108] also observed a decrease in the temperature of the eye until approximately 3 min after an injection of lidocaine buffered with sodium bicarbonate in the corneal nerve, which was associated with pain-related behaviours. When calves were surgically castrated without local anaesthetic, it was detected a slight decrease in the temperature of the eye within the first 3 min as well, but not in calves castrated with anaesthesia. [106]. Martin et al. [103] did not find a decrease in the orbital temperature right after cautery disbudding, with cornual nerve blocking. Bergamasco et al. [109] observed a drop in the mean temperature of the eye immediately after castration (without pain control) and sham castration. These results suggest that the decrease in the temperature of the ocular region can be related to both stress and pain responses. When calves were disbudded with caustic paste, the difference in the maximum eye temperature was not observed right after the procedure. However, the mean horn bud temperature increased due to local inflammation, but not over 12 h after the paste application [110]. This study investigated the efficacy of an oral analgesic (meloxicam) as a pain mitigator for caustic paste disbudding, but the results suggested that it had a limited influence [110]. Kleinhenz et al. [102] compared a different disbudding technique (carbon dioxide laser scalpel) from the traditional hot-iron disbudding. The goal was to provide a less painful procedure, which was not true in this pilot study. IRT images were taken with a thermography camera (Fluke TiX580, Fluke Corp.) from the surface of the disbudded site to check for differences between treatments; no statistical differences were found in the maximum or minimum temperature [102]. Although pain can be prolonged for weeks after the disbudding procedure, IRT seems to not be able to detect differences between burn and uninjured skin in calves [104]. However, it seems more suitable to evaluate acute pain responses or stress responses. In addition, more studies are needed for clarification. The authors suggest for further studies an acclimation period to the procedures and a more restricted control over the animal handling before, during, and after the painful procedures to distinguish between stress- and pain-related responses. Environmental temperature can also affect IRT readings; so, although all papers reviewed have referred that the environmental temperature variation was taken into consideration, its importance should not be diminished.

### 2.4. Image Processing Techniques

There is already some work with image processing techniques to monitor body condition score, lameness, and animal identification in dairy cows [111,112]. However, in calves, the number of papers is still minimal, despite the promising results obtained so far [113,114,115]. 

The calves’ body weight was automatically estimated (mean relative error of 6.50%), measuring body volume, average hip height, and standard deviation of the height distribution in the rear body part with 3D cameras using time-of-flight technology [113]. Stereo cameras were also used with 3D images to estimate the calves’ body weight by indirectly measuring chest and waist girth with a good correlation coefficient (*r* = 0.868) [114]. More recently, Jang et al. [115] obtained high correlation coefficients when estimating Korean cattle weight using both stereo (R^2^ = 0.955) and time-of-flight (R^2^ = 0.956) technology; however, the weight estimation error increased when calves with less than 6 months old were used in the model. Given the present results, estimating calves’ body weight through 3D images can be more complex than in older animals, but it could be a great advantage for the farmer to monitor preweaning efficiency. 

According to Stygar et al. [13], human-animal interaction could be monitored with 3D cameras; however, no studies are currently testing it to the authors’ knowledge. Using machine vision technology, Guo et al. [116] constructed a new method to automatically monitor some calves’ basic behaviours that could be useful for health and welfare assessment; these behaviours include entering and leaving the rest area, remaining stationary, turning around, feeding, and drinking [116]. Automatically monitoring calf behaviours could be a suitable option against classical human observations or even video observations, which are high-cost time-labour activities. 

### 2.5. Heart Rate Monitors

Heart rate variability (HRV) reflects the stress level of mammals by measuring the autonomic stress response and is best quantified by the root mean square of successive differences (RMSSD) in inter-heartbeat intervals [108,116]. HRV is especially useful for investigating the balance between sympathetic and vagal activity and, according to Borell et al. [117], has a great potential for stress and animal welfare assessments. HRV can be measured with heart rate monitors (HRM), and it is used in calves to assess health and welfare problems, concerning that an acclimation period to the monitors is granted. HRMs are usually fitted with a belt tied up behind the calf’s foreleg. Shaving the application site and using ultrasound gel can be used to facilitate electrode contact with the thorax [109]. The HRM usually only stays fitted in the animal during a certain phase of the trial period, around 15 to 60 min [81,109,118,119], in order to record the intended measurement interval, from which data can be directly obtained (Bluetooth [118]) or downloaded after to a computer [109]. Although, according to Stewart et al. [120], HRMs are susceptible to displacement and damage and may change the calf’s normal behaviour, thus, influencing the results. Nevertheless, it is not yet evident if the dairy calves’ HRV better reflects physical activity or emotional states [9]. 

Mohr et al. [119] observed, contrary to linear measures of HRV, that nonlinear measures of HRV were good indicators of stress in calves, either by external factors such as insect harassment or by illness (diarrhoea). Byrd et al. [68] used nonlinear HRV measures with an HRM (Polar H10; Polar Electro Oy, Kempele, Finland) to access disbudding pain; short-term detrended fluctuation analysis varied significantly between control calves and calves disbudded with pain-mitigation (lidocaine and meloxicam), but no differences were obtained with calves disbudded without pain-mitigation. These results limit the utilisation of nonlinear HRV measurements as a pain indicator and suggest that calves disbudded with pain-mitigation may have experienced stress or pain related to the procedure or an effect of pain-mitigating drugs on the autonomic function may have occurred, so further studies are needed for clarification. Stewart et al. [78] observed a significant increase in heart rate (*p* < 0.001) from 5 min before and 5 min after disbudding in calves with and without local anaesthetic. Nevertheless, heart rate stayed elevated longer in calves without local anaesthetic; RMSSD was not significant for any group. Behaviour observations showed higher physical activity in calves disbudded without local anaesthetic (*p* < 0.001). These results are not conclusive on the utilisation of HRV measurements as a pain indicator in calves, so further studies are needed. 

Lürzel et al. [121] used HRM (Polar Electro Oy, Kempele, Finland) to test differences between calves treated with gentle interactions and calves treated with the usual routine, but HRV did not vary with treatment; nonetheless, avoidance distances were lower (*p* = 0.02), and average daily gain was higher (*p* = 0.07) in stroked calves. Furthermore, Stewart et al. [122] did not find significant differences in heart rate between calves treated with positive interactions and calves treated with negative interactions. With HRM (Polar Equine RS800CX Science, Polar Electro UK Ltd., Heathcote Way, Warwick, UK), Clapp et al. [123] observed a significant negative correlation between log RMSSD and time after birth, the calf spent with the mother before separation; on the day of separation: R^2^ = −0.68, *p* = 0.03 and 3 days after separation R^2^ = −0.35, *p* = 0.045. Lv et al. [118] observed an effect of group size on calves’ heart rate, associated with an increase in stress from competition in larger groups. Furthermore, an increase in heart rate (*p* < 0.01) was associated with negative emotions upon milk deprivation in calves at one month of age [124]. 

Generally, HRMs are suitable equipment to monitor HRV measures and are easy to fit calves in a research context. However, it seems that HRV is not a good indicator of pain in calves and may be more related to stressful situations, but more studies are still needed for clarification. 

### 2.6. Ruminal Boluses

The current knowledge on the capacity of ruminal boluses to monitor calves’ health and welfare is limited. Currently, two different bolus types have been tested, one that measures temperature and another that measures pH. A radiofrequency temperature monitoring bolus has the advantage of obtaining several temperature measurements of the core body temperature without further manipulation of the animal besides the bolus application. For example, biothermal rumen boluses proved to be effective at measuring heifers’ core body temperature variations upon a febrile state in response to a viral-bacterial respiratory challenge, using rectal temperature as a control measure [125]. However, Knauer et al. [126] used temperature monitoring bolus (Bella Ag LLC, Loveland, CO, USA) to detect pyrexia (RT of 39.5 °C) in calves with ages comprised between 1 and 11 wk old, but the association with RT was low (concordance correlation coefficient of 0.29, Se of 29%, and Sp of 93%; an increase in Se (71%) was achieved with a cut-off point of 38.75 °C rumen temperature). In addition, the water and milk intake did not significantly affect rumen temperature [126], as it can do in adult cattle [127], probably because milk is usually close to body temperature when fed and can also bypass the rumen through the oesophageal groove directly to the abomasum, which can also happen with water. 

A ruminal bolus with a radio transmission pH sensor to monitor rumen acidosis in dairy cows was developed, and the results had a good correlation with manual sampling (r = 0.986, *p* < 0.01; YCOW-S; DKK-TOA Yamagata, Yamagata, Japan [128]. Later, the same boluses were used in calves to evaluate the effect of dietary forage and calf starter on rumen pH [129], which showed that 200 to 400 g/d of hay starting at 4 wk of age had a beneficial effect on the rumen health; however, a reference method was not used to check the results. In another study, calves from 1 to 12 wk of age fed on a diet containing a starter and chopped straw had suboptimal pH levels (<5.8) [130]. Continuous rumen pH monitoring during weaning and early post-weaning can provide valuable information to improve diet composition and hence the calves’ performance. One limitation of these boluses is linked to the internal battery’s lifetime (2.5 months mean when continuously transmitting every 10 min [128]. Data logging boluses (Well Cow Limited, Roslin, UK) were used in heifers to monitor temperature and pH every 15 min; this bolus was designed for adult cattle, so the application had some constraints [131]. Nevertheless, a positive association between rumen temperature and BRD was observed, but not with rumen pH; a significant variation in temperature and pH across the day was also observed [131].

More validation studies in calves are needed, but the technology seems to have the potential to detect alterations in the core body temperature caused by an infectious event. The calf’s age, and thus, the rumen development, the type of bolus, and the circadian rhythm are some aspects that should be taken into account for future validation studies. 

### 2.7. Location Devices

Behaviour patterns and calves’ preferences can be monitored with global position system (GPS), similar to what has been done with adult grazing cows [48] and other species, such as sheep [132] and goats [133]. GPS investigation in animals is mainly used to monitor the geographic position of wild and farm animals in pasture-based systems. In pasture-based farms, especially in beef cattle farms, calves sometimes go missing, representing an economic loss for the farmer and a welfare problem that GPS devices could aid. Nevertheless, GPS can also be used inside barns, though there are some limitations regarding the accuracy and high energy consumption compared with alternatives such as video cameras [134]. Alawneh et al. [135] fitted eleven weaned Holstein calves with GPS data loggers (I got-U GT600, Mobile Action Technology Inc., Taipei, Taiwan) for two weeks during spring in a paddock with 105 × 30 m to describe and analyse movement patterns. Among other observations, Alawneh et al. [135] found that those calves were most active during the afternoon and at night. Individual or group observations could greatly interest calf welfare evaluations, such as substrate preference and a specific position in the barn or paddock due to thermal conditions. Occhiuto et al. [136] fitted ultra-wideband sensors (Sewio Leonardo iMU tags) in calves to measure individual movement patterns under normal management conditions. The study corroborated the existence of different personality traits within a group of calves and that some calves may be more predictable than others. In addition, more active calves are more exploratory and less likely to choose a favourite spot within the pen but more prone to show displacement behaviours. Calves with these traits are probably more resilient to changes in the environment but may need a larger space to fulfil their needs [136]. Studies regarding cow-calf contact systems are gaining interest in dairy farming; thus, the utilisation of location devices could provide intel about maternal-filial relationships, spatial preferences, and social interactions [137] in this type of calf-raising system. 

### 2.8. Sound Analysis Systems

Animals use vocalisations to communicate and express themselves in a vast array of situations. Interpreting these vocalisations can give us information about the animal’s welfare state [138,139]. Calves vocalise when in pain or stress [140], for example, at disbudding [141], illness [142] or separation from their mother [143]. Nevertheless, calves also vocalise to communicate in neutral stress environments, intra- and interspecies. In farm animals: cattle [144], sheep [145], goats [146], horses [147], pigs [148], and chickens [149], the sound vocalisation pattern, waveform, or fundamental frequencies have been evaluated with microphones, video cameras, sound analysis software, and specific algorithms. Green et al. [150] used spectrographic analysis of dairy cows’ vocalisations upon cow-calf separation to assess emotional stress. Vocalisations may be associated with prolonged hunger or stress derived from feeding management. Thomas et al. [151] found that calves vocalised more often and with a higher fundamental frequency when fed milk 2 times per day (5 L in 24 h) than every 4 h (8 L in 24 h; Beyer dynamic MCE 86 © microphone). On the other hand, De Paula Vieira et al. [29] recorded calf vocalisations using the Beyerdynamic MCE 86 (C) microphone (Farmingdale, NY, USA) but found no differences between milk-restricted calves (10% of BW) and *ad libitum* fed calves, both in an AMFS. One possible explanation for different results relies on the manual feeding in Thomas et al. [151] versus the automatic feeding in De Paula Vieira et al. [29]. In addition, the calf may be compelled to vocalise more depending on the caretaker’s behaviour [29].

Coughing sounds are one of the best clinical signs of BRD in calves [152], and its automatic detection has already been tested [142,153,154]. The cough sounds have distinguished acoustic features, such as amplitude, frequency, and duration [153], which makes it possible to be recorded and identified with a specific algorithm for calves since using algorithms from other species may not be adequate [142]. Vandermeulen et al. [142] developed an automated calf cough monitor and tried to detect BRD in 62 calves. The algorithm had a precision of 87.5%, an Sp of 99.2% and a Se of 50.3%. The coughing pattern was also affected by husbandry management, increasing in periods of more human activity [142]. It is common to observe calves coughing when a straw is added to the calves’ pens without any relation to respiratory diseases. So, that is an aspect to be regarded in the algorithm’s development. Another algorithm was developed with better robustness, improving practical implementation; however, Se was lower (41.4%), but precision (94.2%) and Sp (99.9%) were higher [154]. BRD detection in calves using cough patterns automatic monitoring shows good potential but requires further development to increase Se. Moreover, it is important to note that this method does not allow for individual detection, limiting its commercial application. 

Sound analysis was also used to monitor rumination in calves [155,156,157] and heifers [154,158] by capturing audio recordings of rumen motility with a neck-collar sensor. Only one sensor was found in the researched literature (Hr-Tag, SCR Engineers Ltd., Netarya, Israel). According to the results obtained so far, this sensor has a better performance with heifers than with calves. The Hr-Tag sensor was compared with the chewing halters method in 3 yr old Jersey heifers, and it had very good correlations [158]. Burfeind et al. [155] obtained a good correlation in rumination time between the measurements taken by the sensor and visual observations in 2 mo old calves (r = 0.89, *n* = 5 calves with 3 measurements of 2 h periods) and 9 mo old heifers (r = 0.88, *n* = 5 heifers with 3 measurements of 2 h periods), but the correlations in calves and heifers younger than 9 mo were weaker. The authors also found that suckling did not affect the sensor readings. Rodrigues et al. [157] found a moderate correlation between the sensor rumination time and visual observations (ρ = 0.702, *p* < 0.001) in 32 zebu calves with 77 ± 4 d of age during the pre- and post-weaning period. The sensor seems to overestimate rumination time mainly because it recorded non-existent rumination [157]. Monitoring the daily rumination pattern in 2 to 35 d old calves allowed us to observe some interesting results, such as a marked decrease in rumination around milk feeding time; however, it is important to note that a standard gold method was not used in this study [156]. The sensor showed a significant positive correlation with rumen pH (r = 0.98, *p* < 0.001, *n* = 8 Simmental calves) and a negative correlation with total volatile fatty acids (r = −0.90, *p* < 0.001, *n* = 9), acetate (r = −0.85, *p* < 0.01, *n* = 9) and with propionate (r = −0.90, *p* < 0.001, *n* = 9), which could be linked to higher absorption of volatile fatty acids with the consequent increase in the pH [159]. Monitoring rumination in young calves can provide data to improve nutritional parameters and control rumen development, which can help in the weaning management and identify calves at risk of rumen disorders. The results published so far reveal that the Hr-Tag sensor has the potential to monitor rumination, but it still needs to be validated in young ruminants. Three-dimensional accelerometers were also used to monitor rumination activity [56,67], but the accuracy of both technologies has not been tested simultaneously. 

### 2.9. Multi-Technological Approach 

Although simple and straightforward management practices can be easier to implement and require less technology, the combination of different technologies could provide a more accurate predictive model to detect ill calves before clinical diagnosis and a more accurate measurement of the pain level upon painful procedures; however, there are still few papers incorporating a multiple-technological approach. The main difficulty relies on finding a proper algorithm to integrate the huge amount of data originating from different sources. Martin et al. [103] evaluated the efficacy of bupivacaine liposome suspension as a pain controller during and after dehorning. Although there were no differences between treatments in the IRT of the ocular region, pressure mat gait analysis showed that dehorned calves had an increased gait distance compared to sham dehorned calves (*p* = 0.04). This is the first time pressure mat gait analysis that has been used with calves, so further studies are needed to validate this system as a pain indicator properly. Theurer et al. [160] combined the utilisation of 3D accelerometers (GP1 SENSR, Reference LLC, Elkader, IA, USA) and a remote triangulation device (Ubisense Series 7000 Compact Tag; Ubisense, Denver, CO, USA) to evaluate the effect of oral meloxicam on the calves’ behaviour post-dehorning. The meloxicam calves spent more time at the feed bunk during the 7 days trial, but the control calves spent more time at the hay feeding station during days 0 and 1. Feeding was not recorded, so whether they were eating or not at the feeding stations was not established. Meloxicam calves spent more time lying down than control calves for 5 days post-treatment [159]. Unfortunately, the number of lying bouts was not recorded, which could have provided further intel about the experiment. Castrated 6 mo old Angus bull calves injected with flunixin meglumine and lidocaine had increased dry matter intake, per day, compared with nonmedicated calves. However, noncastrated calves medicated also had a higher dry matter intake [70].

The integration of AMFS with different technologies is the most researched combination. AMFSs are very popular among dairy farmers, and calves voluntarily visit the milk feeder several times a day until weaning, making it ideal for routine data collection. A partial-weight scale attached to an AMFS was used to successfully monitor daily calves’ weight (r = 0.99) [161], informing the farmer about calves’ growth and loss of weight due to health or managing problems. This system was also used to control the weight gain of calves with different personality traits [42]. This system has the advantage of providing measures every day; however, it can only work until weaning. Three-dimensional cameras are able to measure the calf’s body weight [113,114,115]. However, there is no study combining this technology with the AMFS, despite the inherent practical advantages and the potential to identify calves with suboptimal growth. Lowe et al. [24] combined AMFS, automated water systems, accelerometers, and IRT technologies to predict NCD up to 6 d before clinical signs in calves fed two different milk allowance regimes. 

The lying time, the number of lying bouts and the average bout duration were good predictors of disease. Dependent on the milk allowance regime, calves expressed different behavioural indicators of disease prior to clinical signs observations. For example, calves’ behaviours fed 5 L/d milk, by order of suitability to predict disease events from −6 d to 0 d: increased number of lying bouts (*p* < 0.001) and lying bouts durations (*p* < 0.001) and decreased number of visits to the feeder (visits/day; *p* = 0.002); calves fed 10 L/d milk best suitable indicators of disease were: decreased number of visits to the feeder (visits/day; *p* < 0.001), decrease average lying bout duration (min/bout; *p* = 0.002) and increase in the number of lying bouts (bouts/day; *p* = 0.007) [25]. Average water trough visit duration (min/bout) was not affected by illness. However, total water trough visit duration (min/d) increased in the 5 L/d milk group (*p* < 0.029) and in the 10 L/d milk group (*p* < 0.081), probably related to dehydration caused by diarrhoea, which led to an increase in water consumption, especially in the group with lower milk allowance [25]. 

Calves in a 10 L/d milk regime with BRD also had increased lying time and reduced milk and starter intake 5 d before diagnosis, but with a lower number of lying bouts compared to healthy calves [162]. Drinking speed from healthy and sick calves was not different except on the day of diagnosis. Calves with BRD deviated from their baseline unrewarded visits at −4 d before diagnosis, but a significative interaction between “BRD status” and “day” was found [162]. These results demonstrate that calves with NCD and BRD change their feeding and activity behaviour prior to diagnosis. However, specific indicators seem to be influenced by disease and by milk allowance, which may be related to hunger, even though there is no evidence for that statement. Thus, the utilisation of accelerometers and AMFS information combined have the potential to achieve good results in predicting disease events if combined with appropriate algorithms. For example, a −2-day prediction model based on the individual animal’s own lying and milk feeding behaviour using machine learning algorithms could predict respiratory disease in calves with 0.54 sensitivity and 0.95 specificity [163]. The moderate sensitivity value (ability to correctly identify a disease event) could have been affected by the lower percentage of disease days in the study (only approximately 5% were considered disease days by clinical examination). 

**Figure 2:**
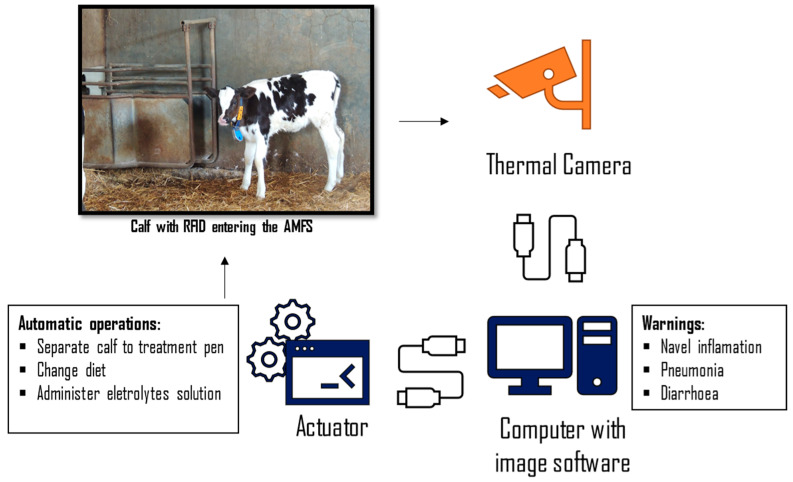
Schematic illustration of a theoretical infrared imaging-based machine vision (IRMV) system applied in a calf pen to provide warnings and automatic operations regarding calves’ health problems.

According to He et al. [164], the technology used to process infrared images automatically is called infrared imaging-based machine vision (IRMV), which is obtained through the intensity of the infrared light emitted or reflected by particular objects. This technology could be of great value to calves and other species. Integrating IRMV into AMFS could provide information about disease onset and alert the caretaker daily (Figure 2). Recently, an algorithm has been validated to process temperatures recorded from IRT automatically [83]. A thermal infrared camera was placed in the AMFS, and developed a capable algorithm to determine the maximum eye and cheek temperatures from the images taken [83]. The images were also analysed manually to compare with the automatic process; the results were positive, with very high correlations, especially eye temperature (R^2^ = 0.99). Machine learning and deep learning are data analysis methods which could significantly increase the real-time monitoring and predictability capacity to detect disease events before clinical symptoms are detected if combined with PDT.

### 2.10. Machine Learning 

Machine learning (ML) is a subfield of artificial intelligence that consists of using computer algorithms with learning capabilities, which means that it can improve based on experience without explicit programming [165]. ML is used in many different fields of expertise—robotics, computer science, telecommunications, finance, physics, biology, etc.—but also aspects of our daily life, such as e-commerce, social networks, and spam e-mails’ filters [166,167,168]. Research on ML has been gaining interest in recent years, despite being used for several decades [169]. 

The main task of ML is making inferences from a sample, so it builds mathematical models with descriptive and predictive characteristics that translate data into valuable information [166,170]. Moreover, ML has a better capacity than classical statistics in searching large databases with different possibilities and determining one hypothesis that best fits the observed data [165]. Another advantage is that ML has techniques less sensitive to spatial autocorrelation and multicollinearity and is also nondependent on classical statistics assumptions (such as homoscedasticity), turning to be more adequate for processing high-dimensional, imbalanced, and nonlinear data [171,172]. A worth mentioning subset technique of ML is deep learning, which is based on artificial neural networks and can be considered an evolution of traditional ML, improving automation, feature selection, and accuracy. In any case, for the purpose of this review, “deep learning” is going to be regarded within the term ML since there are still few significant advances within the scope of the field discussed in the present paper to differentiate both techniques. In animal science, ML techniques have been used mostly in behaviour analysis [116,173] and patterns and traits recognition [174,175,176], in combination with some of the already mentioned technologies [172,177]. Some examples of the most used algorithms are principal component analysis and t-distributed stochastic neighbour embedding (t-SNE) for dimensionality reduction, with k-means for clustering tasks, and decision trees and random forests for classification/regression analysis [172]. 

Guo et al. [116] developed a model (integrated background) that consists of an integration of background-subtraction and inter-frame difference to automatically monitor and recognise dairy calves’ behaviours on a single pen using a machine vision-based method. Activity-based behaviours were automatically recognised with a superior success rate than feeding and drinking behaviours. Another ML method was tested by extracting spatial-temporal features, which was possible by combining a convolutional 3D network and convolution long short-term memory, being able to identify calf’s behaviours with an accuracy of 90.32%. However, physical conditions such as lameness, environmental factors such as light and obstruction by objects, and animal posture may affect the classification results [173]. In another study, several behaviours were accurately identified (ranging from 90.38% to 99.73%) in dairy calves, using collars with a sensor that recorded data from a 3D accelerometer and a 3-axis gyroscope and processed with an ML algorithm—AdaBoost [178]. The same algorithm was used to automatically detect and analyse the eye and cheek regions’ thermal infrared images in calves, despite not being mentioned as an ML technique [83]. 

Machine vision was also used to automatically measure body measurements and weight in calves and heifers, combining ML and 3D cameras with time-of-flight [179] and RGB-D [180] technology. Very recently, three studies have tried to automatically predict diseases in calves using ML, namely NCD [174], BRD [163], and anaplasmosis [181]. Using a random forest learning algorithm, Ma et al. [174] identified six microbial markers in the calves’ gut that could differentiate diarrheic from nondiarrheic calves, which could predict NCD with an accuracy of 84.3%. This study also highlighted the beneficial effects of early gut microbiota variability and stability in the calf’s health and that therapeutic antimicrobials delayed the temporal development of these traits. These results suggest that heat treatment of colostrum and whole milk fed to calves in early life should be revised in terms of its impact on the variability of the calf’s gut microbiota since the general recommendation is to heat treat colostrum before feeding. BRD was predicted in preweaned male calves with a Se of 0.54 and an Sp of 0.95 within a 3-day window [163]. The technologies used were an AMFS and 3D accelerometers to monitor feeding behaviour and activity, using a model that combined moving averages and random forest techniques, which showed better results than the stand-alone models [163]. These results suggest that ML models combined with classical statistics models can provide better results than stand-alone ML models. However, more studies are needed to confirm this statement. Finally, anaplasmosis in calves was predicted using Hr-Tag for rumination time and activity (head movements with an incorporated 3D accelerometer) monitoring and long short-term memory analysis (a recurrent neural network), with an accuracy between 87 and 98% and between 70 and 98% when using rumination time and neck activity, respectively, depending on the day prior to disease confirmation [181]. 

## 3. Conclusions 

Precision technologies contributing to good management and dairy calves’ welfare are rapidly increasing. The AMFS is probably the most researched technology so far, especially in identifying and predicting diseases such as BRD and NCD. It is certain that the calf suffers a change in feeding behaviour with BRD and NCD, but the specific indicators and the number of days prior to clinical signs are not well established. Depending on the aetiology of the disease, different clinical signs can be diagnosed, which could also lead to different expressions of feeding behaviours detected by the AMFS. The accelerometer also has a great expression in studies with dairy calves, providing information about lying and play behaviour, which are associated with health and welfare evaluations. However, the utilisation of accelerometers for rumination and feeding behaviours still needs more validation studies. IRT has been measured in several anatomical regions, but the orbital region seems to be the most sensitive. The utilisation of IRT in calves has increased in recent years, mainly in association with BRD, NCD, omphalitis, and pain evaluations showing good potential, but also needing more investigation to understand the underlying mechanisms and to improve accuracy. Nonetheless, the measurement of the calves’ core body temperature with IRT did not show positive results. In addition, the HRM application needs more research since there is a lack of agreement between studies. It should be mentioned as well that most of the results and applications obtained so far are only possible under research conditions since the commercial application has not been proven effective. 

There are still few studies with other emerging technologies in calves’ science, such as 3D cameras and ruminal bolus, but the results so far are promising. Microphones have multiple applications (vocalisations, coughs, and rumination), and with more validation studies and proper algorithms, they can also have a commercial application to provide alerts of calves at risk. The integration of multiple sensors, wearables, and stationery with machine learning algorithms may provide the necessary to increase commercial application and thus improve calves’ welfare. Finally, it is important to note that none of these technologies can replace the role of human skills and knowledge that remain essential for providing a range of human actions, including animal handling, observation, daily care, monitoring animal health, responding to emergencies and decision-making.

## 4. Challenges for the Future 

There is still a lot to understand about calves’ raising systems, and technology providing precise and constant information can significantly aid our knowledge. An integration system with the AMFS and other technologies, such as accelerometers, IRT, 3D cameras, or a partial-weight scale (for example), should be researched. This system could provide accurate daily information about the calf’s development and well-being. Research should also automatically process this information to provide early alarms about the calves’ condition. One problem is linked to the fact that most of these technologies are best fitted in group-housed calves. 

Usually, after separation from the dam, the calf is housed in a single pen until a few days of life, which may vary according to the farm’s protocol. During this period, there are concerning problems that may compromise the calf’s well-being; so, in the author’s opinion, there is a considerable need to research solutions for this specific period. Feeding and lying behaviours are strong indicators of calves’ health and welfare, with the capacity to predict diseases before clinical signs are precepted by the caretaker. IRT and calves’ growth and body condition combined with those behaviours could provide the needed information to reduce dairy calf morbidity and mortality and improve welfare. The technological application in welfare studies and the integration of appropriate mathematical models to analyse the large data set originating from these sensors gave the opportunity to study the calf needs and desires at the individual level. We strongly believe that future studies with PLF will go to provide essential knowledge to greatly increase calves’ welfare at an individual level and not only at the group level.

## Data Availability

Not applicable.

## References

[1] Pickett H. (2014). Farm Animal Welfare: Past, Present and Future.

[2] Tzanidakis C., Simitzis P., Panagakis P., García Márquez F.P., Lev B. (2023). Systems: Improving Sustainability and Efficiency of Animal Production. Sustainability: Cases and Studies in Using Operations Research and Management Science Methods.

[3] Ponnampalam E.N., Holman B.W.B. (2022). Sustainability II: Sustainable Animal Production and Meat Processing. Lawrie’s Meat Science.

[4] Morrone S., Dimauro C., Gambella F., Cappai M.G. (2022). Industry 4.0 and Precision Livestock Farming (PLF): An up to Date Overview across Animal Productions. Sensors.

[5] Odintsov Vaintrub M., Levit H., Chincarini M., Fusaro I., Giammarco M., Vignola G. (2021). Review: Precision livestock farming, automats and new technologies: Possible applications in extensive dairy sheep farming. Animal.

[6] Knight C.H. (2020). Review: Sensor techniques in ruminants: More than fitness trackers. Animal.

[7] Berckmans D. (2014). Precision livestock farming technologies for welfare management in intensive livestock systems Precision livestock farming: Biology meets technology. Rev. Sci. Tech..

[8] Halachmi I., Guarino M. (2016). Editorial: Precision livestock farming: A ‘per animal’ approach using advanced monitoring technologies. Animal.

[9] Costa J.H.C., Cantor M.C., Neave H.W. (2020). Symposium review: Precision technologies for dairy calves and management applications. J. Dairy Sci..

[10] Eckelkamp E.A. (2019). Invited Review: Current state of wearable precision dairy technologies in disease detection. Appl. Anim. Sci..

[11] Wathes C.M., Kristensen H.H., Aerts J.M., Berckmans D. (2008). Is precision livestock farming an engineer’s daydream or nightmare, an animal’s friend or foe, and a farmer’s panacea or pitfall?. Comput. Electron. Agric..

[12] Bewley J. Precision Dairy Farming: Advanced Analysis Solutions for Future Profitability. Proceedings of the The First North American Conference on Precision Dairy Management.

[13] Stygar A.H., Gómez Y., Berteselli G.V., Dalla Costa E., Canali E., Niemi J.K., Llonch P., Pastell M. (2021). A Systematic Review on Commercially Available and Validated Sensor Technologies for Welfare Assessment of Dairy Cattle. Front. Vet. Sci..

[14] Sun D., Webb L., van der Tol P.P.J., van Reenen K. (2021). A Systematic Review of Automatic Health Monitoring in Calves: Glimpsing the Future From Current Practice. Front. Vet. Sci..

[15] Scoley G., Gordon A., Morrison S.J. (2019). The effect of calf jacket usage on performance, behaviour and physiological responses of group-housed dairy calves. Animal.

[16] Uetake K. (2013). Newborn calf welfare: A review focusing on mortality rates. Anim. Sci. J..

[17] Lombard J., Urie N., Garry F., Godden S., Quigley J., Earleywine T., McGuirk S., Moore D., Branan M., Chamorro M. (2020). Consensus recommendations on calf- and herd-level passive immunity in dairy calves in the United States. J. Dairy Sci..

[18] Donovan G.A., Dohoo I.R., Montgomery D.M., Bennett F.L. (1998). Associations between passive immunity and morbidity and mortality in dairy heifers in Florida, USA. Prev. Vet. Med..

[19] Cramer M.C., Stanton A.L. (2015). Associations between health status and the probability of approaching a novel object or stationary human in preweaned group-housed dairy calves. J. Dairy Sci..

[20] Shecaira C.L., Seino C.H., Bombardelli J.A., Reis G.A., Fusada E.J., Azedo M.R., Benesi F.J. (2018). Using thermography as a diagnostic tool for omphalitis on newborn calves. J. Therm. Biol..

[21] Boccardo A., Sala G., Ferrulli V., Pravettoni D. (2021). Cut-off values for predictors associated with outcome in dairy calves suffering from neonatal calf diarrhea. A retrospective study of 605 cases. Livest. Sci..

[22] Hyde R.M., Green M.J., Sherwin V.E., Hudson C., Gibbons J., Forshaw T., Vickers M., Down P.M. (2020). Quantitative analysis of calf mortality in Great Britain. J. Dairy Sci..

[23] Knauer W.A., Godden S.M., Dietrich A., Hawkins D.M., James R.E. (2018). Evaluation of applying statistical process control techniques to daily average feeding behaviors to detect disease in automatically fed group-housed preweaned dairy calves. J. Dairy Sci..

[24] Lowe G.L., Sutherland M.A., Waas J.R., Schaefer A.L., Cox N.R., Stewart M. (2019). Physiological and behavioral responses as indicators for early disease detection in dairy calves. J. Dairy Sci..

[25] Lowe G.L., Sutherland M.A., Waas J.R., Cox N.R., Schaefer A.L., Stewart M. (2021). Effect of milk allowance on the suitability of automated behavioural and physiological measures as early disease indicators in calves. Appl. Anim. Behav. Sci..

[26] Fraser D. (2008). Understanding animal welfare. Acta Vet. Scand..

[27] Oca M.A. (2021). Temperature Asymmetries in Facial Areas as Indicators of Affective State in Dairy Calves and Horses. Ph.D. Thesis.

[28] Morrison J., Renaud D.L., Churchill K.J., Costa J.H.C., Steele M.A., Winder C.B. (2021). Predicting morbidity and mortality using automated milk feeders: A scoping review. J. Dairy Sci..

[29] De Paula Vieira A., Guesdon V., de Passillé A.M., von Keyserlingk M.A.G., Weary D.M. (2008). Behavioural indicators of hunger in dairy calves. Appl. Anim. Behav. Sci..

[30] Rosenberger K., Costa J.H.C., Neave H.W., von Keyserlingk M.A.G., Weary D.M. (2017). The effect of milk allowance on behavior and weight gains in dairy calves. J. Dairy Sci..

[31] Prado M.E., Wilkerson J., Schneider L.G., Krawczel P.D. (2021). Influence of milk feeding levels and calf housing on subsequent performance of Holstein heifers. JDS Commun..

[32] Khan M.A., Bach A., Weary D.M., von Keyserlingk M.A.G. (2016). Invited review: Transitioning from milk to solid feed in dairy heifers. J. Dairy Sci..

[33] Hostiou N., Fagon J., Chauvat S., Turlot A., Kling-Eveillard F., Boivin X., Allain C. (2017). Impact of precision livestock farming on work and human-animal interactions on dairy farms. A review. Biotechnol. Agron. Société Environ..

[34] Svensson C., Jensen M.B. (2007). Short Communication: Identification of Diseased Calves by Use of Data from Automatic Milk Feeders. J. Dairy Sci..

[35] Borderas T.F., Rushen J., von Keyserlingk M.A.G., de Passillé A.M.B. (2009). Automated measurement of changes in feeding behavior of milk-fed calves associated with illness. J. Dairy Sci..

[36] Knauer W.A., Godden S.M., Dietrich A., James R.E. (2017). The association between daily average feeding behaviors and morbidity in automatically fed group-housed preweaned dairy calves. J. Dairy Sci..

[37] Sutherland M.A., Lowe G.L., Huddart F.J., Waas J.R., Stewart M. (2018). Measurement of dairy calf behavior prior to onset of clinical disease and in response to disbudding using automated calf feeders and accelerometers. J. Dairy Sci..

[38] Cantor M.C., Renaud D.L., Costa J.H.C. (2021). Nutraceutical intervention with colostrum replacer: Can we reduce disease hazard, ameliorate disease severity, and improve performance in preweaned dairy calves?. J. Dairy Sci..

[39] Conboy M.H., Winder C.B., Medrano-Galarza C., LeBlanc S.J., Haley D.B., Costa J.H.C., Steele M.A., Renaud D.L. (2021). Associations between feeding behaviors collected from an automated milk feeder and disease in group-housed dairy calves in Ontario: A cross-sectional study. J. Dairy Sci..

[40] Conboy M.H., Winder C.B., Cantor M.C., Costa J.H.C., Steele M.A., Medrano-Galarza C., von Konigslow T.E., Kerr A., Renaud D.L. (2022). Associations between Feeding Behaviors Collected from an Automated Milk Feeder and Neonatal Calf Diarrhea in Group Housed Dairy Calves: A Case-Control Study. Animals.

[41] Sharpe K.T., Heins B.J. (2021). Growth, health, and economics of dairy calves fed organic milk replacer or organic whole milk in an automated feeding system. JDS Commun..

[42] Carslake C., Occhiuto F., Vázquez-Diosdado J.A., Kaler J. (2022). Indication of a personality trait in dairy calves and its link to weight gain through automatically collected feeding behaviours. Sci. Rep..

[43] Giannetto C., Cerutti R.D., Scaglione M.C., Arfuso F., Pennisi M., Giudice E., Piccione G., Zumbo A. (2023). Real-Time Measurement of the Daily Total Locomotor Behavior in Calves Reared in an Intensive Management System for the Possible Application in Precision Livestock Farming. Vet. Sci..

[44] Riaboff L., Shalloo L., Smeaton A.F., Couvreur S., Madouasse A., Keane M.T. (2022). Predicting livestock behaviour using accelerometers: A systematic review of processing techniques for ruminant behaviour prediction from raw accelerometer data. Comput. Electron. Agric..

[45] Koelsch R.K., Aneshansley D.J., Butler W.R. (1994). Analysis of Activity Measurement for Accurate Oestrus Detection in Dairy Cattle. J. Agric. Eng. Res..

[46] Benaissa S., Tuyttens F.A.M., Plets D., Trogh J., Martens L., Vandaele L., Joseph W., Sonck B. (2020). Calving and estrus detection in dairy cattle using a combination of indoor localization and accelerometer sensors. Comput. Electron. Agric..

[47] Lovarelli D., Bacenetti J., Guarino M. (2020). A review on dairy cattle farming: Is precision livestock farming the compromise for an environmental, economic and social sustainable production?. J. Clean. Prod..

[48] Riaboff L., Couvreur S., Madouasse A., Roig-Pons M., Aubin S., Massabie P., Chauvin A., Bédère N., Plantier G. (2020). Use of Predicted Behavior from Accelerometer Data Combined with GPS Data to Explore the Relationship between Dairy Cow Behavior and Pasture Characteristics. Sensors.

[49] Chapa J.M., Maschat K., Iwersen M., Baumgartner J., Drillich M. (2020). Accelerometer systems as tools for health and welfare assessment in cattle and pigs—A review. Behav. Process..

[50] Pereira G.M., Heins B.J., O’Brien B., McDonagh A., Lidauer L., Kickinger F. (2020). Validation of an ear tag–based accelerometer system for detecting grazing behavior of dairy cows. J. Dairy Sci..

[51] de Passillé A.M., Jensen M.B., Chapinal N., Rushen J. (2010). Technical note: Use of accelerometers to describe gait patterns in dairy calves. J. Dairy Sci..

[52] Bonk S., Burfeind O., Suthar V.S., Heuwieser W. (2013). Technical note: Evaluation of data loggers for measuring lying behavior in dairy calves. J. Dairy Sci..

[53] Swartz T.H., Findlay A.N., Petersson-Wolfe C.S. (2017). Short communication: Automated detection of behavioral changes from respiratory disease in pre-weaned calves. J. Dairy Sci..

[54] Swartz T.H., Schramm H.H., Petersson-Wolfe C.S. (2020). Short Communication: Association between neonatal calf diarrhea and lying behaviors. Vet. Anim. Sci..

[55] Hixson C.L., Krawczel P.D., Caldwell J.M., Miller-Cushon E.K. (2018). Behavioral changes in group-housed dairy calves infected with Mannheimia haemolytica. J. Dairy Sci..

[56] Roland L., Schweinzer V., Kanz P., Sattlecker G., Kickinger F., Lidauer L., Berger A., Auer W., Mayer J., Sturm V. (2018). Technical note: Evaluation of a triaxial accelerometer for monitoring selected behaviors in dairy calves. J. Dairy Sci..

[57] Studds M.J., Deikun L.L., Sorter D.E., Pempek J.A., Proudfoot K.L. (2018). Short communication: The effect of diarrhea and navel inflammation on the lying behavior of veal calves. J. Dairy Sci..

[58] Belaid M.A., Rodríguez-Prado M., Rodríguez-Prado D.V., Chevaux E., Calsamiglia S. (2020). Using behavior as an early predictor of sickness in veal calves. J. Dairy Sci..

[59] Finney G., Gordon A., Scoley G., Morrison S.J. (2018). Validating the IceRobotics IceQube tri-axial accelerometer for measuring daily lying duration in dairy calves. Livest. Sci..

[60] Ahloy-Dallaire J., Espinosa J., Mason G. (2018). Play and optimal welfare: Does play indicate the presence of positive affective states?. Behav. Process..

[61] Luu J., Johnsen J.F., de Passillé A.M., Rushen J. (2013). Which measures of acceleration best estimate the duration of locomotor play by dairy calves?. Appl. Anim. Behav. Sci..

[62] Größbacher V., Bučková K., Lawrence A.B., Špinka M., Winckler C. (2020). Discriminating spontaneous locomotor play of dairy calves using accelerometers. J. Dairy Sci..

[63] Größbacher V., Lawrence A.B., Winckler C., Špinka M. (2020). Negative play contagion in calves. Sci. Rep..

[64] Gardaloud N.R., Guse C., Lidauer L., Steininger A., Kickinger F., Öhlschuster M., Auer W., Iwersen M., Drillich M., Klein-Jöbstl D. (2022). Early Detection of Respiratory Diseases in Calves by Use of an Ear-Attached Accelerometer. Animals.

[65] Goharshahi M., Azizzadeh M., Lidauer L., Steininger A., Kickinger F., Öhlschuster M., Auer W., Klein-Jöbstl D., Drillich M., Iwersen M. (2021). Monitoring selected behaviors of calves by use of an ear-attached accelerometer for detecting early indicators of diarrhea. J. Dairy Sci..

[66] Duthie C.A., Bowen J.M., Bell D.J., Miller G.A., Mason C., Haskell M.J. (2021). Feeding behaviour and activity as early indicators of disease in pre-weaned dairy calves. Animal.

[67] Reynolds M.A., Borchers M.R., Davidson J.A., Bradley C.M., Bewley J.M. (2019). Technical note: An evaluation of technology-recorded rumination and feeding behaviors in dairy heifers. J. Dairy Sci..

[68] Byrd C.J., Craig B.A., Eicher S.D., Radcliffe J.S., Lay D.C. (2019). Short communication: Assessment of disbudding pain in dairy calves using nonlinear measures of heart rate variability. J. Dairy Sci..

[69] Reedman C.N., Duffield T.F., DeVries T.J., Lissemore K.D., Karrow N.A., Li Z., Winder C.B. (2020). Randomized control trial assessing the efficacy of pain control strategies for caustic paste disbudding in dairy calves younger than 9 days of age. J. Dairy Sci..

[70] Nordi W.M., Marti S., Gellatly D., Meléndez D.M., González L.A., McAllister T.A., Fierheller E.E., Caulkett N.A., Janzen E., Schwartzkopf-Genswein K.S. (2019). Effect of preemptive flunixin meglumine and lidocaine on behavioral and physiological indicators of pain post-band and knife castration in 6-mo-old beef calves. Livest. Sci..

[71] Röder M., Heuwieser W., Borchardt S., Plenio J.L., Palme R., Sutter F. (2022). The effect of transdermal flunixin meglumine on blood cortisol levels in dairy calves after cautery disbudding with local anesthesia. J. Dairy Sci..

[72] Heinrich A., Duffield T.F., Lissemore K.D., Millman S.T. (2010). The effect of meloxicam on behavior and pain sensitivity of dairy calves following cautery dehorning with a local anesthetic. J. Dairy Sci..

[73] Coetzee J.F., Mosher R.A., KuKanich B., Gehring R., Robert B., Reinbold J.B., White B.J. (2012). Pharmacokinetics and effect of intravenous meloxicam in weaned Holstein calves following scoop dehorning without local anesthesia. BMC Vet. Res..

[74] Currah J.M., Hendrick S.H., Stookey J.M. (2009). The behavioral assessment and alleviation of pain associated with castration in beef calves treated with flunixin meglumine and caudal lidocaine epidural anesthesia with epinephrine. Can. Vet. J..

[75] Olson M.E., Ralston B., Burwash L., Matheson-Bird H., Allan N.D. (2016). Efficacy of oral meloxicam suspension for prevention of pain and inflammation following band and surgical castration in calves. BMC Vet. Res..

[76] Marti S., Meléndez D.M., Pajor E.A., Moya D., Heuston C.E.M., Gellatly D., Janzen E.D., Schwartzkopf-Genswein K.S. (2017). Effect of band and knife castration of beef calves on welfare indicators of pain at three relevant industry ages: II. Chronic pain. J. Anim. Sci..

[77] Rekant S.I., Lyons M.A., Pacheco J.M., Arzt J., Rodriguez L.L. (2016). Veterinary applications of infrared thermography. Am. J. Vet. Res..

[78] Stewart M., Webster J.R., Schaefer A.L., Cook N.J., Scott S.L. (2005). Infrared thermography as a non-invasive tool to study animal welfare. Anim. Welf..

[79] Mota-Rojas D., Pereira A.M.F., Wang D., Martínez-Burnes J., Ghezzi M., Hernández-Avalos I., Lendez P., Mora-Medina P., Casas A., Olmos-Hernández A. (2021). Clinical applications and factors involved in validating thermal windows used in infrared thermography in cattle and river Buffalo to assess health and productivity. Animals.

[80] Scoley G.E., Gordon A.W., Morrison S.J. (2019). Use of thermal imaging in dairy calves: Exploring the repeatability and accuracy of measures taken from different anatomical regions. Transl. Anim. Sci..

[81] Stewart M., Stafford K.J., Dowling S.K., Schaefer A.L., Webster J.R. (2008). Eye temperature and heart rate variability of calves disbudded with or without local anaesthetic. Physiol. Behav..

[82] Schaefer A.L., Cook N.J., Bench C., Chabot J.B., Colyn J., Liu T., Okine E.K., Stewart M., Webster J.R. (2012). The non-invasive and automated detection of bovine respiratory disease onset in receiver calves using infrared thermography. Res. Vet. Sci..

[83] Lowe G., McCane B., Sutherland M., Waas J., Schaefer A., Cox N., Stewart M. (2020). Automated Collection and Analysis of Infrared Thermograms for Measuring Eye and Cheek Temperatures in Calves. Animals.

[84] Campos J.C.D., Passini R., do Nascimento K.F.M. (2021). Thermography and physiology of stress in dairy calves in outdoor holding pens covered with geosynthetics. Rev. Bras. Eng. Agrícola Ambient..

[85] Schaefer A.L., Cook N., Tessaro S.V., Deregt D., Desroches G., Dubeski P.L., Tong A.K.W., Godson D.L. (2004). Early detection and prediction of infection using infrared thermography. Can. J. Anim. Sci..

[86] Schaefer A.L., Cook N.J., Church J.S., Basarab J., Perry B., Miller C., Tong A.K.W. (2007). The use of infrared thermography as an early indicator of bovine respiratory disease complex in calves. Res. Vet. Sci..

[87] Schaefer A., Perry B., Cook N., Miller C., Church J., Tong A., Stenzler A. (2006). Infrared detection and nitric oxide treatment of bovine respiratory disease (BRD). Online J. Vet. Res..

[88] Wisnieski L., Amrine D.E., Renter D.G. (2021). Predictive modeling of bovine respiratory disease outcomes in feedlot cattle: A narrative review. Livest. Sci..

[89] Lowe G., Sutherland M., Waas J., Schaefer A., Cox N., Stewart M. (2019). Infrared thermography—A non-invasive method of measuring respiration rate in calves. Animals.

[90] Steerforth D.-D., Van Winden S. (2018). Development of clinical sign-based scoring system for assessment of omphalitis in neonatal calves. Vet. Rec..

[91] Robinson A.L., Timms L.L., Stalder K.J., Tyler H.D. (2015). Short communication: The effect of 4 antiseptic compounds on umbilical cord healing and infection rates in the first 24 hours in dairy calves from a commercial herd. J. Dairy Sci..

[92] Bell D.J., Macrae A.I., Mitchell M.A., Mason C.S., Jennings A., Haskell M.J. (2020). Comparison of thermal imaging and rectal temperature in the diagnosis of pyrexia in pre-weaned calves using on farm conditions. Res. Vet. Sci..

[93] Cossa S., Calcante A., Oberti R., Sandrucci A. Monitoring Calf Body Temperature by Infrared Thermography: Preliminary Assessment of Environmental Effects. Proceedings of the 24th Congress of Animal Science and Production Association.

[94] Cantor M.C., Goetz H.M., Beattie K., Renaud D.L. (2022). Evaluation of an infrared thermography camera for measuring body temperature in dairy calves. JDS Commun..

[95] Woodrum Setser M.M., Cantor M.C., Costa J.H.C. (2020). A comprehensive evaluation of microchips to measure temperature in dairy calves. J. Dairy Sci..

[96] Diniz Neto H.C., Lombardi M.C., Campos M.M., Lage A.P., Silva R.O.S., Dorneles E.M.S., Lage C.F.A., Carvalho W.A., Machado F.S., Pereira L.G.R. (2021). Effects of vaccination against brucellosis and clostridia on the intake, performance, feeding behavior, blood parameters, and immune responses of dairy heifers calves. J. Anim. Sci..

[97] Winder C.B., Miltenburg C.L., Sargeant J.M., LeBlanc S.J., Haley D.B., Lissemore K.D., Godkin M.A., Duffield T.F. (2018). Effects of local anesthetic or systemic analgesia on pain associated with cautery disbudding in calves: A systematic review and meta-analysis. J. Dairy Sci..

[98] Sutherland M.A., Worth G.M., Dowling S.K., Lowe G.L., Cave V.M., Stewart M. (2020). Evaluation of infrared thermography as a noninvasive method of measuring the autonomic nervous response in sheep. PLoS ONE.

[99] Tschoner T. (2021). Methods for pain assessment in calves and their use for the evaluation of pain during different procedures—A review. Animals.

[100] Kleinhenz M.D., Van Engen N.K., Gorden P.J., Ji J., Walsh P., Coetzee J.F. (2017). Effects of transdermal flunixin meglumine on pain biomarkers at dehorning in calves. J. Anim. Sci..

[101] Scherf L., Kretschmann J., Fischer M., Mielenz N., Möbius G., Getto S., Kaiser M., Müller H., Bittner L., Baumgartner W. (2020). Thermographic monitoring of skin surface temperature associated with hot-iron disbudding in calves. Schweiz Arch Tierheilkd.

[102] Kleinhenz M.D., Curtis A.K., Weeder M.M., Johnson B.T., Springfield D., Lou M., Viscardi A.V., Coetzee J.F. (2021). Evaluation of a carbon dioxide laser scalpel for disbudding Holstein calves: A pilot study. JDS Commun..

[103] Martin M.S., Kleinhenz M.D., Viscardi A.V., Curtis A.K., Johnson B.T., Montgomery S.R., Lou M.E., Coetzee J.F. (2022). Effect of bupivacaine liposome suspension administered as a cornual nerve block on indicators of pain and distress during and after cautery dehorning in dairy calves. J. Dairy Sci..

[104] Adcock S.J.J., Tucker C.B. (2018). The effect of disbudding age on healing and pain sensitivity in dairy calves. J. Dairy Sci..

[105] Stewart M. (2008). Non-Invasive Measurement of Stress and Pain in Cattle Using Infrared Thermography. Ph.D. Thesis.

[106] Stewart M., Verkerk G.A., Stafford K.J., Schaefer A.L., Webster J.R. (2010). Noninvasive assessment of autonomic activity for evaluation of pain in calves, using surgical castration as a model. J. Dairy Sci..

[107] Kleinhenz M.D., Van Engen N.K., Smith J.S., Gorden P.J., Ji J., Wang C., Perkins S.C.B., Coetzee J.F. (2018). The impact of transdermal flunixin meglumine on biomarkers of pain in calves when administered at the time of surgical castration without local anesthesia. Livest. Sci..

[108] Adcock S.J.J., Tucker C.B. (2022). Buffering lidocaine heightens aversion to cornual nerve injections in dairy calves. J. Dairy Sci..

[109] Bergamasco L., Edwards-Callaway L.N., Bello N.M., Mijares S.H., Cull C.A., Rugan S., Mosher R.A., Gehring R., Coetzee J.F. (2021). Unmitigated surgical castration in calves of different ages: Cortisol concentrations, heart rate variability, and infrared thermography findings. Animals.

[110] Karlen K.J., Baier F.S., Odegard S.L., Baumann R.M., Coetzee J.F., Kehoe S.I., Vogel K.D. (2021). Efficacy of oral meloxicam as primary pain mitigation following caustic paste disbudding of three day old Holstein calves. Transl. Anim. Sci..

[111] O’Mahony N., Campbell S., Carvalho A., Krpalkova L., Riordan D., Walsh J. (2019). 3D Vision for Precision Dairy Farming. IFAC-PapersOnLine.

[112] Silva S.R., Araujo J.P., Guedes C., Silva F., Almeida M., Cerqueira J.L. (2021). Precision technologies to address dairy cattle welfare: Focus on lameness, mastitis and body condition. Animals.

[113] Song X., Schutte J., van der Tol P., van Halsema F., Groot Koer-kamp P. Body Measurements of Dairy Calf Using a 3-D Camera in an Automatic Feeding System. Proceedings of the International Conference of Agricultural Engineering.

[114] Yamashita A., Ohkawa T., Oyama K., Ohta C., Niside R., Honda T. (2018). Calf Weight Estimation with Stereo Camera Using Three-Dimensional Successive Cylindrical Model. J. Inst. Ind. Appl. Eng..

[115] Jang D.H., Kim C., Ko Y.G., Kim Y.H. (2020). Estimation of Body Weight for Korean Cattle Using Three-Dimensional Image. J. Biosyst. Eng..

[116] Guo Y., He D., Chai L. (2020). A Machine Vision-Based Method for Monitoring Scene-Interactive Behaviors of Dairy Calf. Animals.

[117] Von Borell E., Langbein J., Després G., Hansen S., Leterrier C. (2007). Heart rate variability as a measure of autonomic regulation of cardiac activity for assessing stress and welfare in farm animals—A review. Physiol. Behav..

[118] Lv J., Zhao X.W., Su H., Wang Z.P., Wang C., Li J.H., Li X., Zhang R.X., Bao J. (2021). Effects of group size on the behaviour, heart rate, immunity, and growth of Holstein dairy calves. Appl. Anim. Behav. Sci..

[119] Mohr E., Langbein J., Nürnberg G. (2002). Heart rate variability: A noninvasive approach to measure stress in calves and cows. Physiol. Behav..

[120] Stewart M., Wilson M.T., Schaefer A.L., Huddart F., Sutherland M.A. (2017). The use of infrared thermography and accelerometers for remote monitoring of dairy cow health and welfare. J. Dairy Sci..

[121] Lürzel S., Münsch C., Windschnurer I., Futschik A., Palme R., Waiblinger S. (2015). The influence of gentle interactions on avoidance distance towards humans, weight gain and physiological parameters in group-housed dairy calves. Appl. Anim. Behav. Sci..

[122] Stewart M., Shepherd H.M., Webster J.R., Waas J.R., McLeay L.M., Schütz K.E. (2013). Effect of previous handling experiences on responses of dairy calves to routine husbandry procedures. Animal.

[123] Clapp J.B., Croarkin S., Dolphin C., Lyons S.K. (2015). Heart rate variability: A biomarker of dairy calf welfare. Anim. Prod. Sci..

[124] Lv J., Li J., Wang C., Zhao P., Bi Y., Zhang X., Yi R., Li X., Bao J. (2018). Positive or negative emotion induced by feeding success or failure can affect behaviors, heart rate and immunity of suckling calves. Physiol. Behav..

[125] Kayser W.C., Carstens G.E., Washburn K.E., Welsh T.H., Lawhon S.D., Reddy S.M., Pinchak W.E., Chevaux E., Skidmore A.L. (2019). Effects of combined viral-bacterial challenge with or without supplementation of Saccharomyces cerevisiae boulardii strain CNCM I-1079 on immune upregulation and DMI in beef heifers. J. Anim. Sci..

[126] Knauer W.A., Godden S.M., McDonald N. (2016). Technical note: Preliminary evaluation of an automated indwelling rumen temperature bolus measurement system to detect pyrexia in preweaned dairy calves. J. Dairy Sci..

[127] Ipema A.H., Goense D., Hogewerf P.H., Houwers H.W.J., van Roest H. (2008). Pilot study to monitor body temperature of dairy cows with a rumen bolus. Comput. Electron. Agric..

[128] Sato S., Mizuguchi H., Ito K., Ikuta K., Kimura A., Okada K. (2012). Technical note: Development and testing of a radio transmission pH measurement system for continuous monitoring of ruminal pH in cows. Prev. Vet. Med..

[129] Kim Y.H., Toji N., Kizaki K., Kushibiki S., Ichijo T., Sato S. (2016). Effects of dietary forage and calf starter on ruminal pH and transcriptomic adaptation of the rumen epithelium in Holstein calves during the weaning transition. Physiol. Genom..

[130] van Niekerk J.K., Middeldorp M., Guan L.L., Steele M.A. (2021). Preweaning to postweaning rumen papillae structural growth, ruminal fermentation characteristics, and acute-phase proteins in calves. J. Dairy Sci..

[131] Tomczak D.J., Samuelson K.L., Jennings J.S., Richeson J.T. (2019). Oral hydration therapy with water and bovine respiratory disease incidence affects rumination behavior, rumen pH, and rumen temperature in high-risk, newly received beef calves. J. Anim. Sci..

[132] Leu S.T., Quiring K., Leggett K.E.A., Griffith S.C. (2021). Consistent behavioural responses to heatwaves provide body condition benefits in rangeland sheep. Appl. Anim. Behav. Sci..

[133] Lazarus D.D., Opperman P.A., Sirdar M.M., Wolf T.E., van Wyk I., Rikhotso O.B., Fosgate G.T. (2021). Improving foot-and-mouth disease control through the evaluation of goat movement patterns within the FMD protection zone of South Africa. Small Rumin. Res..

[134] Shepley E., Lensink J., Vasseur E. (2020). Cow in Motion: A review of the impact of housing systems on movement opportunity of dairy cows and implications on locomotor activity. Appl. Anim. Behav. Sci..

[135] Alawneh J., Barreto M., Bome K., Soust M. (2020). Description of behavioral patterns displayed by a recently weaned cohort of healthy dairy calves. Animals.

[136] Occhiuto F., Vázquez-Diosdado J.A., Carslake C., Kaler J. (2022). Personality and predictability in farmed calves using movement and space-use behaviours quantified by ultra-wideband sensors. R. Soc. Open Sci..

[137] Pearson C., Filippi P., Lush L., González L.A. (2021). Automated behavioural monitoring allows assessment of the relationships between cow and calf behaviour and calves’ survivability and performance. Appl. Anim. Behav. Sci..

[138] Grandin T. (2001). Cattle vocalizations are associated with handling and equipment problems at beef slaughter plants. Appl. Anim. Behav. Sci..

[139] Manteuffel G., Puppe B., Schön P.C. (2004). Vocalization of farm animals as a measure of welfare. Appl. Anim. Behav. Sci..

[140] Prunier A., Mounier L., Le Neindre P., Leterrier C., Mormède P., Paulmier V., Prunet P., Terlouw C., Guatteo R. (2013). Identifying and monitoring pain in farm animals: A review. Animal.

[141] Caray D., de Boyer des Roches A., Frouja S., Andanson S., Veissier I. (2015). Hot-iron disbudding: Stress responses and behavior of 1- and 4-week-old calves receiving anti-inflammatory analgesia without or with sedation using xylazine. Livest. Sci..

[142] Vandermeulen J., Bahr C., Johnston D., Earley B., Tullo E., Fontana I., Guarino M., Exadaktylos V., Berckmans D. (2016). Early recognition of bovine respiratory disease in calves using automated continuous monitoring of cough sounds. Comput. Electron. Agric..

[143] Rushen J., Wright R., Johnsen J.F., Mejdell C.M., de Passillé A.M. (2016). Reduced locomotor play behaviour of dairy calves following separation from the mother reflects their response to reduced energy intake. Appl. Anim. Behav. Sci..

[144] Schnaider M., Heidemann M., Silva A., Taconeli C., Molento C. (2021). Vocalization and other behaviors as indicators of emotional valence: The case of cow-calf separation and reunion in beef cattle. J. Vet. Behav..

[145] Bishop J.C., Falzon G., Trotter M., Kwan P., Meek P.D. Sound Analysis and Detection, and the Potential for Precision Livestock Farming—A Sheep Vocalisation Case Study. Proceedings of the 1st Asian-Australiasian Conference on Precision Pastures and Livestock Farming.

[146] Silveira L., Antunes L., Silva S., Ferreira D., Soares S., Pinto M., Lebre P., Dionísio I., Serralheiro P. (2020). Reparação do nervo laríngeo recorrente—Estudo experimental Repair of the recurrent laryngeal nerve: Experimental study. Rev. Port. Cir..

[147] Pond R.L., Darre M.J., Scheifele P.M., Browning D.G. (2010). Characterization of equine vocalization. J. Vet. Behav. Clin. Appl. Res..

[148] Herbst C.T., Nishimura T., Garcia M., Migimatsu K., Tokuda I.T. (2021). Effect of Ventricular Folds on Vocalization Fundamental Frequency in Domestic Pigs (Sus scrofa domesticus). J. Voice.

[149] Fontana I., Tullo E., Scrase A., Butterworth A. (2016). Vocalisation sound pattern identification in young broiler chickens. Animal.

[150] Green A.C., Lidfors L.M., Lomax S., Favaro L., Clark C.E.F. (2021). Vocal production in postpartum dairy cows: Temporal organization and association with maternal and stress behaviors. J. Dairy Sci..

[151] Thomas T.J., Weary D.M., Appleby M.C. (2001). Newborn and 5-week-old calves vocalize in response to milk deprivation. Appl. Anim. Behav. Sci..

[152] Lowie T., Van Leenen K., Jourquin S., Pas M.L., Bokma J., Pardon B. (2022). Differences in the association of cough and other clinical signs with ultrasonographic lung consolidation in dairy, veal, and beef calves. J. Dairy Sci..

[153] Ferrari S., Piccinini R., Silva M., Exadaktylos V., Berckmans D., Guarino M. (2010). Cough sound description in relation to respiratory diseases in dairy calves. Prev. Vet. Med..

[154] Carpentier L., Berckmans D., Youssef A., Berckmans D., van Waterschoot T., Johnston D., Ferguson N., Earley B., Fontana I., Tullo E. (2018). Automatic cough detection for bovine respiratory disease in a calf house. Biosyst. Eng..

[155] Burfeind O., Schirmann K., von Keyserlingk M.A.G., Veira D.M., Weary D.M., Heuwieser W. (2011). Technical note: Evaluation of a system for monitoring rumination in heifers and calves. J. Dairy Sci..

[156] Lopreiato V., Minuti A., Piccioli-Cappelli F., Vailati-Riboni M., Britti D., Trevisi E., Morittu V.M. (2018). Daily rumination pattern recorded by an automatic rumination-monitoring system in pre-weaned calves fed whole bulk milk and ad libitum calf starter. Livest. Sci..

[157] Rodrigues J.P.P., Pereira L.G.R., do Carmo Diniz Neto H., Lombardi M.C., de Assis Lage C.F., Coelho S.G., Sacramento J.P., Machado F.S., Tomich T.R., Maurício R.M. (2019). Technical note: Evaluation of an automatic system for monitoring rumination time in weaning calves. Livest. Sci..

[158] Byskov M.V., Schulze A.K.S., Weisbjerg M.R., Markussen B., Nørgaard P. (2014). Recording rumination time by a rumination monitoring system in Jersey heifers fed grass/clover silage and hay at three feeding levels. J. Anim. Sci..

[159] Lopreiato V., Vailati-Riboni M., Morittu V.M., Britti D., Piccioli-Cappelli F., Trevisi E., Minuti A. (2020). Post-weaning rumen fermentation of Simmental calves in response to weaning age and relationship with rumination time measured by the Hr-Tag rumination-monitoring system. Livest. Sci..

[160] Theurer M.E., White B.J., Coetzee J.F., Edwards L.N., Mosher R.A., Cull C.A. (2012). Assessment of behavioral changes associated with oral meloxicam administration at time of dehorning in calves using a remote triangulation device and accelerometers. BMC Vet. Res..

[161] Cantor M.C., Pertuisel C.H., Costa J.H.C. (2020). Technical note: Estimating body weight of dairy calves with a partial-weight scale attached to an automated milk feeder. J. Dairy Sci..

[162] Cantor M.C., Costa J.H.C. (2022). Daily behavioral measures recorded by precision technology devices may indicate bovine respiratory disease status in preweaned dairy calves. J. Dairy Sci..

[163] Bowen J.M., Haskell M.J., Miller G.A., Mason C.S., Bell D.J., Duthie C.A. (2021). Early prediction of respiratory disease in preweaning dairy calves using feeding and activity behaviors. J. Dairy Sci..

[164] He Y., Deng B., Wang H., Cheng L., Zhou K., Cai S., Ciampa F. (2021). Infrared machine vision and infrared thermography with deep learning: A review. Infrared Phys. Technol..

[165] Mitchell T.M. (1997). Machine Learning.

[166] Alpaydin E. (2010). Introduction to Machine Learning.

[167] LeCun Y., Bengio Y., Hinton G. (2015). Deep learning. Nature.

[168] El Naqa I., Murphy M.J. (2015). What is machine learning. Machine Learning in Radiation Oncology.

[169] Katamreddy S., Riordan D., Doody P. Artificial Calf Weaning Strategies and the Role of Machine Learning: A Review. Proceedings of the 2017 28th Irish Signals and Systems Conference (ISSC).

[170] Lokhorst C., De Mol R.M., Kamphuis C. (2019). Invited review: Big Data in precision dairy farming. Animal.

[171] Machado G., Mendoza M.R., Corbellini L.G. (2015). What variables are important in predicting bovine viral diarrhea virus? A random forest approach. Vet. Res..

[172] Valletta J.J., Torney C., Kings M., Thornton A., Madden J. (2017). Applications of machine learning in animal behaviour studies. Anim. Behav..

[173] Qiao Y., Guo Y., Yu K., He D. (2022). C3D-ConvLSTM based cow behaviour classification using video data for precision livestock farming. Comput. Electron. Agric..

[174] Ma T., Villot C., Renaud D., Skidmore A., Chevaux E., Steele M., Guan L.L. (2020). Linking perturbations to temporal changes in diversity, stability, and compositions of neonatal calf gut microbiota: Prediction of diarrhea. ISME J..

[175] Sadeghi M., Banakar A., Khazaee M., Soleimani M. (2015). An Intelligent Procedure for the Detection and Classification of Chickens Infected by Clostridium Perfringens Based on their Vocalization. Rev. Bras. Ciência Avícola.

[176] Denholm S.J., Brand W., Mitchell A.P., Wells A.T., Krzyzelewski T., Smith S.L., Wall E., Coffey M.P. (2020). Predicting bovine tuberculosis status of dairy cows from mid-infrared spectral data of milk using deep learning. J. Dairy Sci..

[177] Neethirajan S. (2020). The role of sensors, big data and machine learning in modern animal farming. Sens. Bio-Sens. Res..

[178] Carslake C., Vázquez-Diosdado J.A., Kaler J. (2020). Machine Learning Algorithms to Classify and Quantify Multiple Behaviours in Dairy Calves Using a Sensor: Moving beyond Classification in Precision Livestock. Sensors.

[179] Nir O., Parmet Y., Werner D., Adin G., Halachmi I. (2018). 3D Computer-vision system for automatically estimating heifer height and body mass. Biosyst. Eng..

[180] Weales D., Moussa M., Tarry C. (2021). A robust machine vision system for body measurements of beef calves. Smart Agric. Technol..

[181] Teixeira V.A., Lana A.M.Q., Bresolin T., Tomich T.R., Souza G.M., Furlong J., Rodrigues J.P.P., Coelho S.G., Gonçalves L.C., Silveira J.A.G. (2022). Using rumination and activity data for early detection of anaplasmosis disease in dairy heifer calves. J. Dairy Sci..

